# Diagnostic accuracy of pulmonary host inflammatory mediators in the exclusion of ventilator-acquired pneumonia

**DOI:** 10.1136/thoraxjnl-2014-205766

**Published:** 2014-10-08

**Authors:** Thomas P Hellyer, Andrew Conway Morris, Daniel F McAuley, Timothy S Walsh, Niall H Anderson, Suveer Singh, Paul Dark, Alistair I Roy, Simon V Baudouin, Stephen E Wright, Gavin D Perkins, Kallirroi Kefala, Melinda Jeffels, Ronan McMullan, Cecilia M O'Kane, Craig Spencer, Shondipon Laha, Nicole Robin, Savita Gossain, Kate Gould, Marie-Hélène Ruchaud-Sparagano, Jonathan Scott, Emma M Browne, James G MacFarlane, Sarah Wiscombe, John D Widdrington, Ian Dimmick, Ian F Laurenson, Frans Nauwelaers, A John Simpson

**Affiliations:** 1 Institute of Cellular Medicine, Medical School, Newcastle University, Newcastle upon Tyne, UK; 2 MRC Centre for Inflammation Research, University of Edinburgh, Edinburgh, UK; 3 Department of Anaesthesia, University of Cambridge, Cambridge Biomedical Campus, Cambridge, UK; 4 Centre for Infection and Immunity, Health Sciences Building, Queen's University Belfast, Belfast, UK; 5 Regional Intensive Care Unit, Royal Victoria Hospital, Belfast, UK; 6 Centre for Population Health Sciences, University of Edinburgh, Medical School, Edinburgh, UK; 7 Intensive Care Unit, Chelsea and Westminster Hospital, Imperial College London, London, UK; 8 Institute of Inflammation and Repair, University of Manchester, Manchester Academic Health Sciences Centre & Intensive Care Unit, Salford Royal NHS Foundation Trust, Greater Manchester, UK; 9 Integrated Critical Care Unit, Sunderland Royal Hospital, Sunderland, UK; 10 Intensive Care Unit, Royal Victoria Infirmary, Newcastle upon Tyne, UK; 11 Intensive Care Unit, Freeman Hospital, Newcastle upon Tyne, UK; 12 University of Warwick and Heart of England NHS Foundation Trust, Coventry, UK; 13 Intensive Care Unit, Royal Infirmary of Edinburgh, Edinburgh, UK; 14 Newcastle Clinical Trials Unit, William Leech Building, Medical School, Newcastle University, Newcastle upon Tyne, UK; 15 Department of Medical Microbiology, Kelvin Building, The Royal Hospitals, Belfast, UK; 16 Intensive Care Unit, Lancashire Teaching Hospitals NHS Foundation Trust, Preston, UK; 17 Intensive Care Unit, Countess of Chester NHS Trust, Chester, UK; 18 Public Health Laboratory, Heart of England NHS Foundation Trust, Birmingham, UK; 19 Public Health England & Newcastle upon Tyne Hospitals NHS Foundation Trust, Freeman Hospital, Newcastle upon Tyne, UK; 20 Bioscience Centre (West Wing), International Centre for Life, Newcastle University, Newcastle upon Tyne, UK; 21 Department of Clinical Microbiology, Royal Infirmary of Edinburgh, Edinburgh, UK; 22 Becton Dickinson Biosciences, Erembodegem (Aalst), Belgium

**Keywords:** Pneumonia

## Abstract

**Background:**

Excessive use of empirical antibiotics is common in critically ill patients. Rapid biomarker-based exclusion of infection may improve antibiotic stewardship in ventilator-acquired pneumonia (VAP). However, successful validation of the usefulness of potential markers in this setting is exceptionally rare.

**Objectives:**

We sought to validate the capacity for specific host inflammatory mediators to exclude pneumonia in patients with suspected VAP.

**Methods:**

A prospective, multicentre, validation study of patients with suspected VAP was conducted in 12 intensive care units. VAP was confirmed following bronchoscopy by culture of a potential pathogen in bronchoalveolar lavage fluid (BALF) at >10^4^ colony forming units per millilitre (cfu/mL). Interleukin-1 beta (IL-1β), IL-8, matrix metalloproteinase-8 (MMP-8), MMP-9 and human neutrophil elastase (HNE) were quantified in BALF. Diagnostic utility was determined for biomarkers individually and in combination.

**Results:**

Paired BALF culture and biomarker results were available for 150 patients. 53 patients (35%) had VAP and 97 (65%) patients formed the non-VAP group. All biomarkers were significantly higher in the VAP group (p<0.001). The area under the receiver operator characteristic curve for IL-1β was 0.81; IL-8, 0.74; MMP-8, 0.76; MMP-9, 0.79 and HNE, 0.78. A combination of IL-1β and IL-8, at the optimal cut-point, excluded VAP with a sensitivity of 100%, a specificity of 44.3% and a post-test probability of 0% (95% CI 0% to 9.2%).

**Conclusions:**

Low BALF IL-1β in combination with IL-8 confidently excludes VAP and could form a rapid biomarker-based rule-out test, with the potential to improve antibiotic stewardship.

**Key messages**

**What is the key question?**

- Ventilator-acquired pneumonia (VAP) is notoriously difficult to diagnose clinically, so this multicentre study aimed to determine whether VAP could be rapidly and accurately excluded by quantifying host biomarkers.

**What is the bottom line?**

- Low concentrations of interleukin-1 beta and interleukin-8 effectively exclude VAP using a system that yields results within 6 h.

**Why read on?**

- To our knowledge, this is the first study to validate the effective, rapid exclusion of VAP using host proteins, paving the way for future trials assessing whether these markers can improve antibiotic stewardship in the intensive care unit.

## Introduction

Antibiotic resistance has been increasing rapidly, making antibiotic stewardship a priority for healthcare systems globally. Patients admitted to the intensive care unit (ICU) receive a significant burden of antibiotics.^1^ Ventilator-acquired pneumonia (VAP) occurs in 10%–20% of the ICU population.^2^ VAP poses a dilemma for clinicians seeking to improve antibiotic stewardship. The diagnosis of VAP is challenging and pulmonary infection is confirmed in only approximately 30% of patients with suspected VAP.^3^ ^4^ Despite this, since VAP is associated with significant mortality and morbidity, and because significant delays in appropriate treatment have been linked to increases in mortality,^5^ patients are often treated with antibiotics from the moment of initial suspicion. This is compounded by the fact that conventional microbiology culture and sensitivity results typically take up to 72 h to return to clinicians.

Novel biomarker-based diagnostic techniques, if suitably accurate and rapid, would offer a significant change in the clinical information available at the time of suspected infection and could reduce unnecessary antibiotic use. However, to our knowledge, no protein biomarkers showing promise in initial derivation studies have gone on to yield confirmatory diagnostic utility in multicentre validation studies.

In a single-centre derivation cohort, mediators of the host inflammatory response measured in bronchoalveolar lavage (BAL) fluid from patients with suspected VAP demonstrated potential as biomarkers for the exclusion of pneumonia. In particular, low concentrations of BAL fluid interleukin (IL)-1β appeared to be able to rule out VAP effectively.^6^ IL-8 and the neutrophil proteases matrix metalloproteinase-8 (MMP-8), MMP-9 and human neutrophil elastase (HNE) also showed promise in excluding VAP.^7^ We, therefore, conducted a pragmatic multicentre validation study of these five BAL fluid biomarkers in patients with suspected VAP.

## Methods

### Study design and participants

We conducted a prospective, multicentre, observational study in 12 general UK ICUs, with screening performed on week days between February 2012 and February 2013. A wide case mix of medical, surgical and trauma patients was represented. Patients were eligible if they were aged 18 years or more and if they had been endotracheally intubated and mechanically ventilated for at least 48 h. VAP was suspected if the patient had new or worsening alveolar shadowing on chest radiograph (CXR) and if at least 2 of the following criteria were present: purulent tracheal secretions; temperature <35°C or >38°C; or a blood white cell count <4×10^9^/L or >11×10^9^/L. Patients were excluded based on criteria predicting poor tolerance of bronchoscopy and BAL: PaO_2_ <8 kPa on FiO_2_ >0.7; positive end-expiratory pressure >15 cm H_2_O; peak airway pressure >35 cm H_2_O; heart rate >140 bpm; mean arterial pressure <65 mm Hg; bleeding diathesis (including platelet count <20×10^9^/L or international normalised ratio >3); or intracranial pressure >20 mm Hg. Patients were also excluded if the ICU clinician responsible for the patient's care considered the procedure to be unsafe, if the patient had a previous BAL as part of this study or if consent/assent was not obtained.

Consent or assent was obtained according to approved procedures for incapacitated adults. The study was approved by the appropriate research ethics committees within the National Research Ethics Service (England and Northern Ireland (11/NE/0242), Scotland (11/SS/0089)). Study monitoring and oversight were provided by Newcastle Clinical Trials Unit.

### Procedures

Participants underwent a protocolised bronchoscopy and BAL performed by either the clinical team or local investigators. In summary, BAL was performed in a region of the lung corresponding to an area of new alveolar infiltrate on CXR. If multiple areas were involved, the BAL was performed in a segment or subsegment from which purulent secretions were visualised. If the CXR changes were extensive or there was doubt over which segment to lavage, the posterior segment of the right lower lobe was sampled.^8^ Patients received 100% oxygen and sedation with or without paralysis according to the clinical team's preference. The bronchoscope was gently wedged in the segment to be lavaged. The first 20 mL saline instillate was aspirated and discarded. Thereafter, three 40 mL aliquots of saline were instilled, aspirated and pooled.

A 2 mL aliquot of BAL fluid was sent to a National Health Service or Public Health England microbiology laboratory for semiquantitative culture. BAL fluid was handled according to a standard operating procedure in accordance with the UK Standards for Microbiological Investigation.^9^ VAP was confirmed by growth of potential pathogens at >10^4^ colony forming units per millilitre (cfu/mL), the value of which as a reference test in VAP has been discussed extensively elsewhere.^2^ Sterile specimens or growth below this threshold identified the ‘non-VAP’ group. The remaining BAL fluid was centrifuged at 700 g for 10 min. The supernatant was aspirated and stored frozen for subsequent biomarker quantification, which was performed at a single site (Newcastle University) at the end of study recruitment. Therefore, routine clinical care was provided to all patients involved, without access to biomarker results.

Biomarkers in BAL fluid were measured by cytometric bead array (CBA) and analysed using an Accuri C6 flow cytometer (Becton Dickinson Biosciences, New Jersey, USA). Results are generated within approximately 4 h. Levels of IL-1β, IL-8, MMP-8, MMP-9 and HNE were determined in BAL fluid in a 5-bead multiplex according to the manufacturer's instructions. CBA kits for IL-1β and IL-8 were commercially available, whereas CBA kits for MMP-8, MMP-9 and HNE were custom-made for the study. All samples were measured in dilutions of 1:5, 1:50 and 1:500. Samples which fell below the standard range in the 1:5 dilution were repeated using undiluted BAL fluid. CBA was carried out by a single trained investigator, who was unaware of the culture results, in a separate laboratory to which BAL fluid cultures were performed.

Clinical data were collected on ICU length of stay, duration of mechanical ventilation, time to ICU and hospital discharge, in-hospital mortality, ICU mortality, admission category (medical or surgical), Acute Physiology and Chronic Health Evaluation II score on admission, use of renal replacement therapy, use of vasopressors, use of corticosteroids and whether criteria for acute respiratory distress syndrome were met.^10^ Data were also collected on antibiotic use on day of enrolment and during the preceding 72 h; and antibiotic days and antibiotic-free days (AFD) in the 7 days following BAL.

### Statistical considerations

Sample size was estimated from the derivation study post-test probability (PTP) of VAP using the IL-1β threshold value for exclusion.^6^ Below the threshold, IL-1β excluded VAP with a PTP of 2.8% (95% CI 0.1% to 15.9%). To improve the external validity of this result, a sample size was based on narrowing the 95% CI. We estimated that 24% of patients would have confirmed VAP based on the derivation cohort. A sample size of 140 was estimated to allow a 95% CI for a PTP of 3% to be 0% to 8%, which was judged tight enough for potential clinical use. We, therefore, planned to recruit 160 patients to allow for a dropout rate of approximately 15%.

Statistical analysis was performed using SPSS V.19 (Chicago, Illinois, USA) and R 3.0.0.^11^ Comparisons for non-normally distributed continuous data were made with the Mann–Whitney U test, otherwise with the Student t test. Binary outcomes were analysed using the χ^2^ test.

Since the objective was to validate a rule-out for VAP, the statistical analysis was performed to determine the maximum sensitivity and negative predictive value (NPV). Patients were dichotomised into a VAP group and non-VAP group according to growth in BAL fluid of >10^4^ cfu/mL or ≤10^4^ cfu/mL. Individual biomarkers that were significantly different between groups were analysed by receiver operator characteristic (ROC) procedures. Biomarkers were log_10_ transformed with the addition of a constant of one before being tested in combination. Log_10_ transformed biomarkers were entered into a logistic regression model and ROC curves were constructed from the predictive model. Cut-points for individual biomarkers and combinations were determined by fixing a minimum NPV of 95%. The diagnostic rules, diagnostic performance measures and associated 95% CIs were derived using the OptimalCutpoints library in R3.0.0.^12^

## Results

Four hundred and fifteen patients satisfied criteria for suspected VAP, of whom 248 had exclusion criteria. Of the remaining 167 patients, 150 had paired microbiological culture and biomarker results from BAL fluid and were entered into the analysis. Fifty-three patients (35%) had confirmed VAP and the remaining 97 (65%) patients comprised the non-VAP group. The Standards for Reporting of Diagnostic Accuracy (STARD) diagram is provided at the end of the Results section, after the rationale for the optimal diagnostic test has been described.

The VAP and non-VAP groups were similar in terms of demographics and clinical characteristics (table 1). The VAP group was associated with less use of antibiotics prior to BAL, less use of corticosteroids and a higher proportion of surgical patients, but only the first of these was statistically significant (table 1).

**Table 1:** Patient characteristics.

Characteristic	VAP (N=53)	Non-VAP (N=97)	p Value
Age (years)	56.3±17.7	55.2±17.3	0.48
Male—n (%)	42 (79.2)	69 (71.1)	0.28
Time from ICU admission to consent (days)—median (IQR)*	5 (4–8)	7 (4–11)	0.43
APACHE II score on admission	17.3±6.0	19.1±7.8	0.15
Surgical admission—n (%)	31 (58.5)	41 (42.3)	0.06
Medical admission—n (%)	22 (41.5)	56 (57.7)	0.06
Hospital mortality—n (%)†	19 (38.8)	31 (33)	0.49
Hospital LOS (days)—median (IQR)‡	39 (25–65)	36 (20–51)	0.26
ICU LOS (days)—median (IQR)§	17 (14–34)	17 (11–31)	0.21
Renal replacement therapy—n (%)¶	5 (9.4)	12 (12.5)	0.57
Vasopressors—n (%)†	12 (23.1)	35 (36.5)	0.10
ARDS criteria—n (%)**	8 (16)	19 (21.3)	0.44
Receipt of corticosteroids—n (%)*	7 (13.2)	21 (22.8)	0.16
Antibiotics at time of BAL—n (%)	30 (56.6)	80 (82.5)	0.001
New antibiotics started in 72 hours before BAL—n (%)	8 (15.1)	27 (27.8)	0.08
Antibiotic days in the 7 days post BAL—median (IQR)*	7 (5–7)	6.5 (4–7)	0.43
Antibiotic-free days (AFD) in the 7 days following BAL—median (IQR)*	0 (0–2)	0 (0–2.3)	0.21

Plus/minus values are mean±SD.

Data missing for: *5 patients; †7 patients; ‡21 patients; §2 patients; ¶1 patient and **11 patients.

APACHE II, Acute Physiology and Chronic Health Evaluation II; ARDS, acute respiratory distress syndrome; BAL, bronchoalveolar lavage; ICU, intensive care unit; LOS, length of stay; VAP, ventilator-acquired pneumonia.

The organisms grown at >10^4^ cfu/mL are shown in table 2. Gram-negative bacteria accounted for approximately 60% of identified organisms, Gram-positive organisms 30% and fungi 10%. In the VAP group, 39 patients (73%) had growth of a single micro-organism, 12 (23%) had two micro-organisms and 2 patients had three micro-organisms cultured at >10^4 ^cfu/mL.

**Table 2:** Organisms cultured at >10^4^ cfu/mL

Organism	N
Methicillin-sensitive *Staphylococcus aureus*	14
*Escherichia coli*	8
*Pseudomonas aeruginosa*	7
*Klebsiella pneumoniae*	5
*Haemophilus* spp	6
*Candida* spp	5
*Enterobacter aerogenes*	2
*Enterobacter cloacae*	1
*Acinetobacter* spp	2
Coliform	2
*Moraxella catarrhalis*	2
Upper respiratory flora*	2
*Streptococcus pneumoniae*	2
*Proteus mirabilis*	2
*Serratia marcescens*	1
*Citrobacter koseri*	1
*Stenotrophomonas maltophilia*	1
*Streptococcus pyogenes*	1
Methicillin-resistant *Staphylococcus aureus*	1
*Peptostreptococcus* spp	1
Yeasts	2
*Streptococcus* group C	1

N, the number of patients in whom the micro-organism in question was isolated from bronchoalveolar lavage fluid at >10^4^ cfu/mL.

*In both cases, normal flora growth was in addition to another organism at >10^4^ cfu/mL.

**Figure 1:**
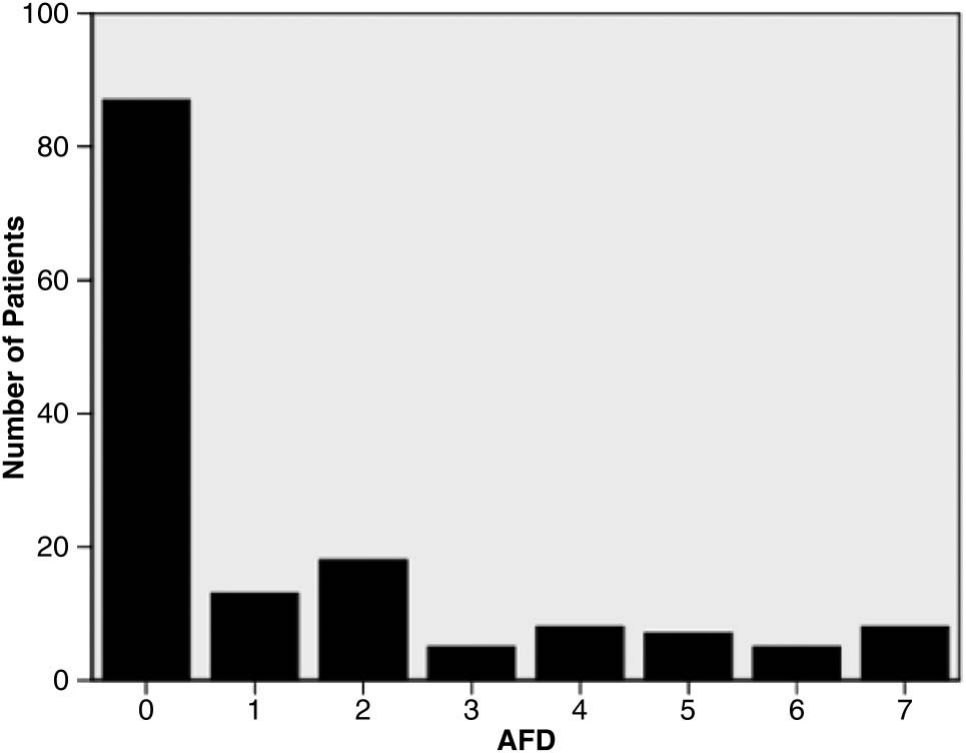
Frequency distribution of AFD in the 7 days following bronchoalveolar lavage. AFD, antibiotic-free days, represented as an integer value.

One hundred and ten patients (73.3%) were receiving antibiotics at the time of BAL, but only 35 patients (23.3%) had a new antibiotic started in the 72 h before BAL, with no significant difference between the VAP and non-VAP groups (p=0.078). In the 7 days following BAL, 57.6% of patients had no AFD, with the median AFD being 0 (IQR 0–2) days. The distribution of AFD in the 7 days following BAL is shown in figure 1.

There were significant differences in the concentrations of IL-1β, IL-8, MMP-8, MMP-9 and HNE in BAL fluid when comparing the VAP and non-VAP groups (p<0.001 for all comparisons) (table 3). Therefore, ROC curves were constructed for all 5 biomarkers to determine diagnostic utility and optimum cut-off points. Selecting cut-points to obtain a minimum NPV of 95% resulted in unacceptably low specificity for IL-8, MMP-8 and HNE (table 4).

**Table 3:** Biomarker concentration

Biomarker	VAP	Non-VAP	p Value	AUROC (95% CI)
IL-1β *pg/mL*	712 (112–1999)	29 (3–184)	<0.001	0.81 (0.74 to 0.88)
IL-8 *pg/mL*	7546 (1987–23 050)	1401 (369–4422)	<0.001	0.74 (0.65 to 0.82)
MMP-8 *ng/mL*	734 (113–2792)	66 (11–325)	<0.001	0.76 (0.68 to 0.84)
MMP-9 n*g/mL*	6840 (1721–22 221)	491 (106–3146)	<0.001	0.79 (0.71 to 0.86)
HNE *ng/mL*	3882 (710–11 183)	349 (96–1473)	<0.001	0.78 (0.70 to 0.85)

BAL fluid biomarker concentrations for VAP and non-VAP groups. Data are expressed as median and IQR.

AUROC, area under the receiver operator characteristic curve; BAL, bronchoalveolar lavage; HNE, human neutrophil elastase; IL, interleukin; MMP, matrix metalloproteinase; VAP, ventilator-acquired pneumonia.

**Table 4:** Individual biomarkers and biomarker combinations

Biomarker/combination *Cut-point*	Sensitivity *(95% CI)*	Specificity *(95% CI)*	PPV *(95% CI)*	NPV *(95% CI)*	+LR *(95% CI)*	−LR *(95% CI)*	PTP (%) *(95% CI)*
IL-1β *17 pg/mL*	96.2% (*87.2 to 99.0)*	43.3% (*33.9 to 53.2)*	0.48 (*0.39 to 0.58)*	0.96 (*0.85 to 0.99)*	1.70 (*1.41 to 2.04)*	0.09 (*0.02 to 0.35)*	4.5 (*1.3 to 5.1)*
IL-8 *382 pg/mL*	98.1% (*90.1 to 99.7)*	24.7% (*17.2 to 34.2)*	0.42 (*0.33 to 0.50)*	0.96 (*0.80 to 0.99)*	1.30 (*1.16 to 1.47)*	0.08 (*0.01 to 0.55)*	4 *(0.7 to 19.5)*
MMP-8 *160 ng/mL*	100% (*93.3 to 100.0)*	5.2% (*1.7 to 11.6)*	0.37 (*0.29 to 0.45)*	1.0 (*0.57 to 1.0)*	1.05 (*1.01 to 1.10)*	0.0 (NE)	0.0 *(0.0 to 0.43)*
MMP-9 *296 ng/mL*	96.2% (*87.0 to 99.5)*	43.4% (*33.3 to 53.7)*	0.48 (*0.38 to 0.58)*	0.96 (*0.85 to 0.97)*	1.70 (*1.41 to 2.04)*	0.09 (*0.02 to 0.35)*	4.5 *(3.9 to 15.3)*
HNE *161 ng/mL*	98.1% (*89.9 to 99.9)*	34.0% (*24.7 to 44.3)*	0.45 (*0.36 to 0.54)*	0.97 (*0.85 to 0.98)*	1.49 (*1.28 to 1.72)*	0.06 (*0.01 to 0.39)*	2.9 *(1.9 to 15.0)*
IL-1β/IL-8	100% (*93*.*2 to 100.0)*	44.3% (*34*.*2 to 54*.*8)*	0.50 (*0*.*39 to 0*.*59)*	1.0 (*0*.*92 to 1*.*0)*	1.80 (*1*.*50 to 2*.*15)*	0.0 (NE)	0.0 (*0.0 to 9.2)*
IL-1β/IL-8/ MMP-9	98.1% (*89*.*9 to 100.0)*	44.3% (*34*.*2 to 54*.*8)*	0.49 (*0*.*39 to 0*.*60)*	0.98 (*0*.*88 to 0.98)*	1.76 (*1*.*47 to 2*.*11)*	0.04 (*0*.*01 to 0*.*30)*	2.3 (*1.5 to 11.9)*
IL-1β/IL-8/MMP-8/MMP-9	100% (*93*.*3 to 100.0)*	46.4% (*36*.*2 to 56*.*8)*	0.51 (*0*.*41 to 0*.*60)*	1.0 (*0*.*92 to 1*.*0)*	1.87 (*1*.*55 to 2*.*24)*	0.0 (NE)	0.0 (*0.0 to 7.8)*
IL-1β/IL-8/MMP-8/MMP- 9/HNE	98.1% (*90*.*0 to 100.0)*	46.4% (*36*.*2 to 56*.*8)*	0.50 (*0*.*40 to 0*.*60)*	0.98 (*0*.*89 to 0.99)*	1.83 (*1*.*52 to 2*.*21)*	0.04 (*0*.*01 to 0*.*29)*	2.2 (*1.4 to 11.5)*

Biomarkers were log_10_ transformed with the addition of a constant of one before fitting into logistic regre**s**sion for combinations of biomarkers. The linear predictor from each logistic regression was used to construct an ROC curve. Performance of each combination represents performance at the specific cut-point on the ROC curve.

HNE, human neutrophil elastase; IL, interleukin; −LR, negative likelihood ratio; +LR, positive likelihood ratio; MMP, matrix metalloproteinase; NE, not estimated; NPV, negative predictive value; PPV, positive predictive value; PTP, post-test probability; ROC, receiver operator characteristic.

Log_10_ transformed biomarkers were highly correlated, with Pearson's correlation coefficients ranging from 0.79 to 0.93. IL-1β was the strongest individual predictor of VAP; therefore, combinations of biomarkers were tested with IL-1β. ROC curves were constructed for combinations and performance at optimal cut-points derived. The top performing biomarker combinations were IL-1β/IL-8/MMP-8/MMP-9 (cut-point −1.7015 on the scale of the linear predictor) with a sensitivity of 100% and a specificity of 46.4%, and IL-1β/IL-8 (cut-point −1.7616 on the scale of the linear predictor) with a sensitivity of 100% and a specificity of 44.3% (table 4).

**Figure 2:**
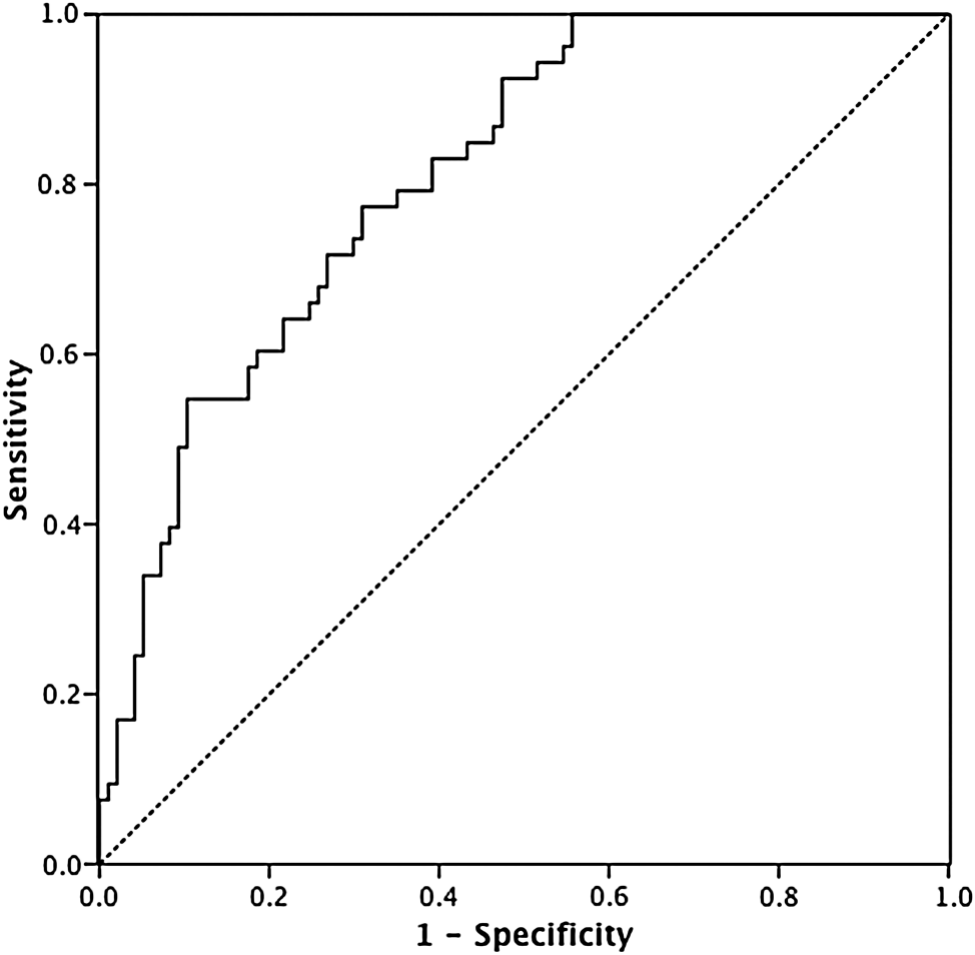
Receiver operator characteristic (ROC) curve of interleukin (IL)-1β and IL-8 combination. ROC curve of linear predictor of IL-1β and IL-8 logistic regression. Area under the ROC curve=0.81 (95% CI 0.74 to 0.88).

The area under the ROC curve (AUROC) for the IL-1β/IL-8 combination was 0.81 (95% CI 0.74 to 0.88) (figure 2). Our aim of achieving a high NPV resulted in a lower specificity; however, either of these biomarker combinations could exclude VAP with a PTP of 0% (95% CI 0% to 7.8% for the 4-biomarker combination; 0% to 9.2% for the 2-biomarker combination). A 2-biomarker combination would have pragmatic advantages over a 4-biomarker combination for future clinical application. As a further validation of the logistic regression model, the 2-biomarker model was applied to the biomarkers from the derivation cohort.^6^ ^7^ The resulting AUROC was 0.85 (95% CI 0.75 to 0.94). Using the same cut-point of −1.7616 yielded a sensitivity of 94.1%, a specificity of 56.4% and an NPV of 96.9%.

**Figure 3:**
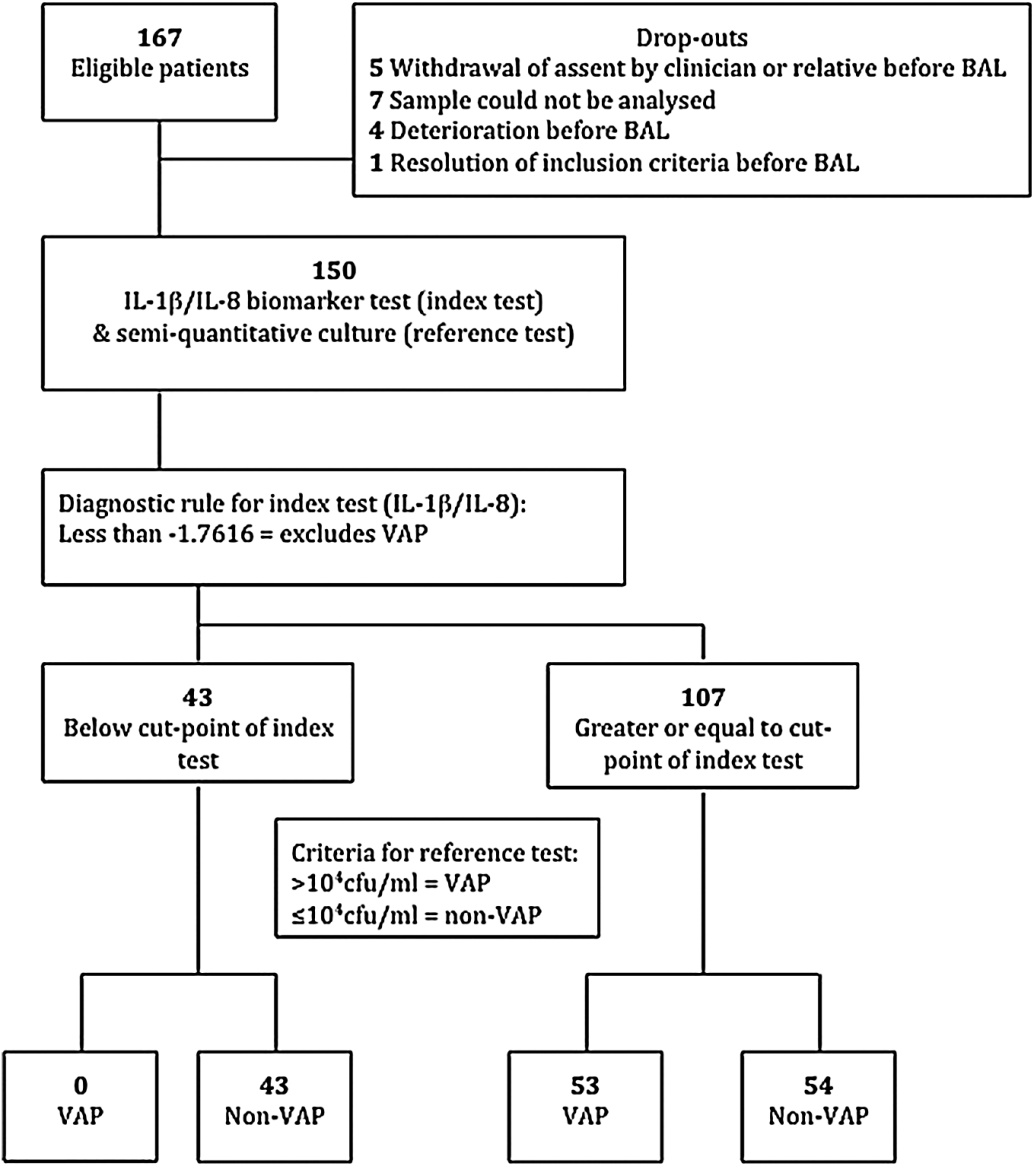
STARD diagram for IL-1β/IL-8 combination. BAL, bronchoalveolar lavage; IL, interleukin; VAP, ventilator-acquired pneumonia; STARD, Standards for Reporting of Diagnostic Accuracy.

The STARD diagram for the study is shown in figure 3.

## Discussion

This multicentre validation cohort from a broad ICU population yielded results very similar to those from our previous single-centre derivation cohort. The optimal cut-offs for IL-1β were also similar in the 2 studies (10 vs 17 pg/mL), the small difference probably being explained by the lower volume of BAL instilled in this study (120 mL vs 200 mL). The data, therefore, potentially provide important information towards the development of new, rapid diagnostic strategies for infection in the ICU. Successful validation of initially promising derivation studies has represented a major hurdle in the development of new diagnostics in the ICU setting. Of the five biomarkers investigated, all were significantly higher in VAP than in the non-VAP group. We demonstrate that IL-1β, in particular, is a powerful biomarker for the exclusion of VAP. Indeed, when the diagnostic utility of combinations of biomarkers was tested, IL-1β was the predominant component of all models. The combination of IL-1β and IL-8 could exclude VAP with an NPV of 1, suggesting this simple combination has significant potential as a rule-out test for VAP. The cut-point was selected with the aim of determining the optimal characteristics for exclusion of VAP. With an NPV of 1 but a positive predictive value of 0.50, the biomarkers reflect the performance of a satisfactory ‘rule-out’ test. Furthermore, this assay typically takes approximately 4 h to perform, yielding a rapid and novel biomarker combination to exclude VAP.

There is growing evidence that short courses of antibiotics for suspected sepsis in the ICU may be safe. In contrast, there is evidence that overuse of antibiotics could be harmful.^13^ Our data suggest that if empirical antibiotics were started in all patients with suspected VAP, they could potentially be discontinued with high confidence within a few hours in response to low IL-1β and IL-8 concentrations in BAL fluid. If clinicians were prepared to change antibiotic prescription based on the rule-out results, we estimate that up to 30% of patients with suspected VAP could have early discontinuation of antibiotics, when they would otherwise be continued.

To our knowledge, no other biomarkers have been shown to be so robust for the exclusion of VAP. The most widely investigated biomarkers in VAP are procalcitonin and type 1 soluble triggering receptor expressed on myeloid cells (sTREM-1), which have been analysed in both BAL fluid and serum.^14–16^ In addition, other biomarkers, including elastin fibres, copeptin, nitrated proteins, serum β-d-glucan, pancreatic stone protein, midregional pro-atrial natriuretic peptide, pentraxin 3, Clara cell protein, leucocyte RNA profiles, leptin, and gene expression, have been investigated.^17–27^ These biomarkers have generally shown inconsistent results, often with poor diagnostic or prognostic utility. The biomarkers analysed in the present study potentially have biological plausibility in that IL-1β and IL-8 are pro-inflammatory cytokines, while MMP-8, MMP-9 and HNE may be released from activated neutrophils during degranulation, and one might expect these mediators to be elevated in regions of lung in which there is active infection.

The diagnostic criteria and method of sampling used have been variable in previous biomarker studies in suspected VAP. Although debate continues over the merits of bronchoscopic and non-bronchoscopic methods for respiratory sampling in terms of clinical outcomes, the need for consistent alveolar sampling is probably of greater importance when measuring biomarkers in suspected VAP. VAP is known to be a patchy process,^8^ and ‘blind’ BAL methods may (at least theoretically) either sample unaffected alveolar regions or, worse, proximal non-alveolar airway. When seeking VAP, it seems logical to sample the alveolar regions of radiologically affected lung. Although one other study did not show any diagnostic utility from IL-1β,^16^ this variance to our findings may be due to sampling by mini-BAL. Our results can only be extrapolated from the protocolised BAL used in this study, and certainly our cut-off values cannot be extrapolated to mini-BAL, for example. Although the practical implications and cost of BAL may be perceived as potential disincentives, BAL is a common procedure in the ICU and the full cost implications can only be considered once the impact on antibiotic prescribing has been determined in future studies.

Our study has a number of potential limitations. As with many ICU studies, exclusion criteria eliminated 60% of patients who met inclusion criteria, potentially reducing the generalisability. A caveat of our reliance on BAL sampling is that some patients are too unstable to tolerate the procedure. We estimate that 67 patients (16%) who met inclusion criteria were excluded on safety grounds precluding bronchoscopy (data not shown).

A further potential limitation is that a large proportion of patients (73.3%) were on antibiotics at the time of BAL, which could potentially have resulted in false negative microbiology and, perhaps, even falsely low biomarker levels. However, far fewer patients had a new antibiotic started in the 72 h before BAL (23.3%) with no significant difference between the VAP and non-VAP groups. Importantly, this was a pragmatic validation study, and excluding patients on antibiotics would have further limited its generalisability. Performing a subanalysis of IL-1β to exclude patients who received new antibiotics in 72 h before BAL (therefore including 69 non-VAP and 46 VAP patients) resulted in an AUROC of 0.78 (95% CI 0.70 to 0.87), suggesting that the effect of antibiotics may not be of great significance.

An additional consideration is that our ‘standard’ for the diagnosis of VAP—potential pathogens at >10^4^ cfu/mL in BAL fluid—is imperfect. If a patient is on an antibiotic at the time of BAL and a pathogen sensitive to that antibiotic is cultured, but at <10^4^ cfu/mL, clinicians may find it hard to be absolutely confident that VAP is excluded. The obvious difficulty here is that the true diagnostic ‘gold standard’ of simultaneous culture and histological examination of infected and inflamed alveolar tissue is impractical. In this setting, quantitative culture generally performs at least as well as other suggested diagnostic modalities, and better than clinical diagnosis without sampling.^2^ ^28^ Taken overall, we can only conclude that our study validates the performance of IL-1β and IL-8 in the specific setting of the protocolised BAL and the definition of VAP used here.

Furthermore, determining the highest achievable sensitivity comes at the cost of specificity. As the specificity decreases, the proportion of non-VAP patients correctly ruled out by the biomarker combination reduces. However, this trade-off is necessary to exclude VAP with the lowest possible PTP, to give the clinician confidence in the rule-out performance and therefore allow early antibiotic discontinuation. We also recognise that a blood marker(s) excluding VAP would be far more desirable than BAL fluid markers, given that some patients may not be suitable for BAL and that there is potential interoperator technical variability in BAL. However, at present, no good blood biomarkers exist in this context.

A further issue concerns the ongoing controversy as to whether *Candida* spp in BAL fluid at >10^4^ cfu/mL are contaminants or potentially pathogenic. We included *Candida* on the basis that, in critically ill patients, clinicians may find it hard to ignore potential pathogens at such high concentrations from an affected region of lung. In any event, isolated growth of *Candida* was rare in our hands. Finally, a diagnostic test is only valuable if it beneficially alters practice, and it remains to be seen whether clinicians will be prepared to alter antibiotic use based on a biomarker test—this requires to be scrutinised formally in the setting of a randomised controlled trial.

In summary, this study confirms that IL-1β effectively excludes VAP when validated in a multicentre study. Performance is improved further by the addition of IL-8, and the combination could form a relatively simple, rapid diagnostic assay to exclude VAP. Biomarker analysis appears to have the potential to improve antibiotic stewardship early in the course of suspected VAP. Whether this concept can lead to effective improvements in antibiotic stewardship remains to be seen and should be the focus of randomised controlled trials.

## References

[1] Vincent JL, Rello J, Marshall J International study of the prevalence and outcomes of infection in intensive care units. JAMA 2009;302:2323–9. 19952319 10.1001/jama.2009.1754

[2] Chastre J, Fagon JY Ventilator-associated pneumonia. Am J Respir Crit Care Med 2002;165:867–903. 11934711 10.1164/ajrccm.165.7.2105078

[3] Meduri GU, Mauldin GL, Wunderink RG Causes of fever and pulmonary densities in patients with clinical manifestations of ventilator-associated pneumonia. Chest 1994;106:221–35. 8020275 10.1378/chest.106.1.221

[4] Fagon JY, Chastre J, Hance AJ Detection of nosocomial lung infection in ventilated patients: use of a protected specimen brush and quantitative culture techniques in 147 patients. Am J Respir Crit Care Med 1988;138:110–16. 10.1164/ajrccm/138.1.110 3144202

[5] Iregui M, Ward S, Sherman G Clinical importance of delays in the initiation of appropriate antibiotic treatment for ventilator-associated pneumonia. Chest 2002;122:262–8. 12114368 10.1378/chest.122.1.262

[6] Conway Morris A, Kefala K, Wilkinson TS Diagnostic importance of pulmonary interleukin-1beta and interleukin-8 in ventilator-associated pneumonia. Thorax 2010;65:201–7. 19825784 10.1136/thx.2009.122291 PMC2866736

[7] Wilkinson TS, Morris AC, Kefala K Ventilator-associated pneumonia is characterized by excessive release of neutrophil proteases in the lung. Chest 2012;142:1425–32. 22911225 10.1378/chest.11-3273

[8] Rouby JJ, Martin De Lassale E, Poete P Nosocomial bronchopneumonia in the critically ill. Histologic and bacteriologic aspects. Am Rev Respir Dis 1992;146:1059–66. 1416397 10.1164/ajrccm/146.4.1059

[9] Health Protection Agency. UK standards for Microbiology investigations. Investigation of Bronchoalveolar Lavage, Sputum and Associated Specimens. Issued by the Standards Unit, Microbiology Services Division, HPA. Issue date 02.08.12.

[10] Bernard GR, Artigas A, Brigham KL Report of the American-European Consensus conference on ARDS: definitions, mechanisms, relevant outcomes and clinical trial coordination. The Consensus Committee. Intensive Care Med 1994;20:225–32. 8014293 10.1007/BF01704707

[11] Team RC R: A language and environment for statistical computing. Vienna, Austria: R Foundation for Statistical Computing, 2013.

[12] Lopez-Ranton M, Rodriguez-Alvarez M Package “OptimalCutpoints.” 2013. http://cran.rproject.org/web/packages/OptimalCutpoints/OptimalCutpoints.pdf

[13] Kett DH, Cano E, Quartin AA Implementation of guidelines for management of possible multidrug-resistant pneumonia in intensive care: an observational, multicentre cohort study. Lancet Infect Dis 2011;11:181–9. 21256086 10.1016/S1473-3099(10)70314-5

[14] Duflo F, Debon R, Monneret G Alveolar and serum procalcitonin: diagnostic and prognostic value in ventilator-associated pneumonia. Anesthesiology 2002;96:74–9. 11753005 10.1097/00000542-200201000-00018

[15] Luyt CE, Combes A, Reynaud C Usefulness of procalcitonin for the diagnosis of ventilator-associated pneumonia. Intensive Care Med 2008;34:1434–40. 18421435 10.1007/s00134-008-1112-x

[16] Gibot S, Cravoisy A, Levy B Soluble triggering receptor expressed on myeloid cells and the diagnosis of pneumonia. N Engl J Med 2004;350:451–8. 14749453 10.1056/NEJMoa031544

[17] el-Ebiary M, Torres A, González J Use of elastin fibre detection in the diagnosis of ventilator associated pneumonia. Thorax 1995;50:14–17. 7886642 10.1136/thx.50.1.14 PMC473697

[18] Seligman R, Papassotiriou J, Morgenthaler NG Copeptin, a novel prognostic biomarker in ventilator-associated pneumonia. Crit Care 2008;12:R11. 18252006 10.1186/cc6780 PMC2374597

[19] Mathy-Hartert M, Damas P, Nys M Nitrated proteins in bronchoalveolar lavage fluid of patients at risk of ventilator-associated bronchopneumonia. Eur Respir J 2000;16:296–301. 10968506 10.1034/j.1399-3003.2000.16b18.x

[20] Heyland D, Jiang X, Day AG Serum β-d-glucan of critically ill patients with suspected ventilator-associated pneumonia: preliminary observations. J Crit Care 2011;26:536.e1–9. 21376516 10.1016/j.jcrc.2011.01.002

[21] Boeck L, Graf R, Eggimann P Pancreatic stone protein: a marker of organ failure and outcome in ventilator-associated pneumonia. Chest 2011;140:925–32. 21835904 10.1378/chest.11-0018

[22] Seligman R, Papassotiriou J, Morgenthaler NG Prognostic value of midregional pro-atrial natriuretic peptide in ventilator-associated pneumonia. Intensive Care Med 2008;34:2084–91. 18523752 10.1007/s00134-008-1173-x

[23] Lin Q, Fu F, Shen L Pentraxin 3 in the assessment of ventilator-associated pneumonia: an early marker of severity. Heart Lung 2013;42:139–45. 23273657 10.1016/j.hrtlng.2012.11.005

[24] Vanspauwen MJ, Linssen CFM, Bruggeman CA Clara cell protein in bronchoalveolar lavage fluid: a predictor of ventilator-associated pneumonia?. Crit Care 2011;15:R14. 21223571 10.1186/cc9418 PMC3222046

[25] Cobb JP, Moore EE, Hayden DL Validation of the riboleukogram to detect ventilator-associated pneumonia after severe injury. Ann Surg 2009;250:531–9. 19730236 10.1097/SLA.0b013e3181b8fbd5 PMC3047595

[26] Parmentier-Decrucq E, Nseir S, Makris D Accuracy of leptin serum level in diagnosing ventilator-associated pneumonia: a case-control study. Minerva Anestesiol 2014;80:39–47. 24107832

[27] Textoris J, Loriod B, Benayoun L An evaluation of the role of gene expression in the prediction and diagnosis of ventilator-associated pneumonia. Anesthesiology 2011;115:344–52. 21796056 10.1097/ALN.0b013e318225ba26

[28] Chastre J, Fagon JY, Bornet-Lecso M Evaluation of bronchoscopic techniques for the diagnosis of nosocomial pneumonia. Am J Respir Crit Care Med 1995;152:231–40. 7599829 10.1164/ajrccm.152.1.7599829

